# Jewish Ethics of Inmate Vaccines Against COVID-19

**DOI:** 10.1007/s11673-023-10331-x

**Published:** 2024-03-01

**Authors:** Tsuriel Rashi

**Affiliations:** School of Communication, Ariel University, 65 Ramat HaGolan St., Ariel, Israel

**Keywords:** Jewish ethics, Inmates vaccinations, COVID-19

## Abstract

**Purpose:**

The COVID-19 pandemic broke out at the end of 2019, and throughout 2020 there were intensive international efforts to find a vaccine for the disease, which had already led to the deaths of some five million people. In December 2020, several pharmaceutical companies announced that they had succeeded in producing an effective vaccine, and after approval by the various regulatory bodies, countries started to vaccinate their citizens. With the start of the global campaign to vaccinate the world’s population against COVID-19, debates over the prioritization of different sections of the population began around the world, but the prison population has generally been absent from these discussions.

**Approach and Findings:**

This article presents the approach of Jewish ethics regarding this issue, that is, that there is a religious and a moral obligation to heal the other and to take care of his or her medical well-being and that this holds true even for a prisoner who has committed a serious crime. Hence, prisoners should be vaccinated according to the same priorities that govern the administration of the vaccine among the general public.

**Originality:**

The originality of the article is in a comprehensive and comparative reference between general ethics and Jewish ethics on a subject that has not yet received the proper attention.

## COVID-19 and the Global Efforts for Vaccination

Vaccinating a population against dangerous infectious diseases, which is a preventive medical service that a country owes its citizens, is based on the recommendations of professionals and health authorities around the world. The basket of recommended vaccinations is updated from time to time according to need.

Vaccination, which has proved to be one of the world’s most important advances in public health, began in 1796 with Edward Jenner’s development of a vaccine against smallpox (Koppaka 1999). Various protocols have since prevented the spread of deadly diseases in an effective and efficient manner. The COVID-19 pandemic has affected tens of millions of people and caused the death of more than five million. The pandemic apparently erupted in December 2019 in the city of Wuhan, China, and by the middle of February 2020 had started to spread, causing panic, which was soon followed by economic crises around the world. In December 2020, the Moderna, Pfizer, and AstraZeneca drug companies announced that they had succeeded in producing vaccines against COVID-19 and received approvals to distribute the vaccines from various regulators, including the FDA in the United States and the European EMA.

The issue of “prisoners’ rights” is on the agenda of many countries in the Western democratic-liberal world. The basic approach is that even behind prison walls, an individual does not lose his rights. Whereas incarceration causes him to lose his freedom it does not negate the rest of his rights, although it may reduce their scope. In practice, there is a built-in tension between the desire to preserve the prisoner’s rights and their actual protection. The COVID-19 pandemic has already put some countries to a moral and ethical test regarding the vaccination of prisoners and detainees around the world.

## Vaccination Against COVID-19 in Prison Systems

There were reports of outbreaks of COVID-19 in prisons in many countries. Responses were highly variable and it is not clear whether public health guidance was informed by the best available evidence (Beaudry, et al. 2020). A “call for urgent action” published in *The Lancet* medical journal in October 2020 noted that in the United States, more than forty of the fifty largest clustered outbreaks occurred in prisons (Macmadu et al. 2020; Siva 2020).^1^ According to Quandt, thirty-nine out of the fifty states did not or seemed not to address the incarcerated population as a priority group at all, neither in their original prioritization plans nor in later updates (Quandt 2020).

As reported by the Marshal Project, by December 2020 one in five prisoners in the United States, which has the highest incarceration rate in the world, had had COVID-19 (Schwartzapfel, Park, and DeMillo 2020). Whereas vaccination priorities and distribution are set by each state, groups such as the American Medical Association (2020) lobbied for inmates to be included in initial rollouts, along with essential workers in the criminal justice sector. The federal prison system inoculates prison employees first (Sequera, et al. 2013).

The United Kingdom, the United States, and most of Europe were among the first to begin their national vaccination programmes against COVID-19, but experts were concerned about the notable absence of prison populations in planning and protocols. The United Kingdom’s Joint Committee on Vaccination and Immunization said that the first priority for vaccines was to prevent deaths and protect health- and social-care staff and systems, with no specific mention of prisons.

Canada, in contrast, started vaccinating older and medically vulnerable federal inmates in the first phase of its vaccine programme, launched at the beginning of 2021 (Correctional Service Canada 2021). Neither the World Health Organization nor the European Union had specific guidelines for when prison populations should be vaccinated, a decision they said should be determined by their member nations (Berger 2021).

This article does not deal with the legal justifications for vaccinating prisoners but presents the ethical arguments for vaccinating prisoners posited in Jewish ethics. One must take as a starting point and understand the meaning of the religious duty to take care of the other’s medical needs in general and the proper ethical treatment of prisoners in particular. In both areas it is not the individual’s right that is in question but a religious and humane obligation.

## The Importance of Life in Jewish Heritage

The serious prohibition against spilling blood underlies human moral awareness. The response and complaint of Cain that he is not his brother’s keeper (Genesis 4:9) called forth the harsh query: “What have you done? The voice of your brother’s blood is crying to me from the ground” (Genesis 4:10). To this, we see in the Midrash, Rabbi Nathan adds: “This teaches us that the blood of his children, grandchildren and all his descendants to the end of time were present and shouting to G-d. This tells us that one person is equal to the entire Creation” (Goldin 1990, ver. 1, ch. 31). Thus, as soon as Noah emerged from the ark, the entire human species was forcefully and sternly commanded to take care not to do harm: “Whoever sheds the blood of man, by man shall his blood be shed, for G-d made man in his own image” (Genesis 9:6). This type of thinking has inspired the major religions around the world and has been realized during the COVID-19 pandemic as well (Bouayed et al. 2021).

Rabbi Meir (second century CE) affirmed the dual import, wherein there is not just a warning against doing harm but also an emphasis on the value of saving:

“Therefore man was created alone in the world, to teach us that anyone who destroys a single soul is as though he destroyed a whole world. And anyone who saves a single soul is as though he maintained the whole world” (Neusner 1991, Sanhedrin 2: 4).

Maimonides wrote that each person, because of his/her uniqueness and the unique trajectory of his/her life, is considered a whole world: “All the inhabitants of the world are created in the image of Adam, the first man, and yet no one person’s face resembles the face of his colleague. Therefore, each person can say: ‘The world was created for me’” (Maimonides 1954, “Laws of Sanhedrin,” 12:3). The decisive acknowledgment in the Mishnah of the singularity of each person made it the source of inspiration for the principles of individualism in eighteenth-century liberalism.^2^ Today, too, the Mishnah affirms not just the rights that each individual is born with but also the obligation incumbent upon every human being to reach out to and save fellow humans in their hour of distress (Skinner 1998; Dunn 1969). Every individual, including a refugee, is a unique human being.

Love of one’s fellow that leads someone to share in another’s pain and to come to his/her aid is one of the values espoused by Judaism. The zenith of love of one’s fellow is expressed in “he who saves one life saves a whole world” (Neusner 1991, Sanhedrin 4: 2).^3^ Thus, it is altogether right and proper to volunteer to help an individual in distress and certainly to offer aid to a group of suffering people. Such help is an expression of the attributes of mercy and peace, which are characteristics ascribed to G-d and to which a person should aspire. Responsibility for the reasonable quality of people’s lives and for making the effort to help them are also fostered by the vision of the prophetic End of Days.

## The Obligation to Save Life and the Priority in Medical Treatment

Many rabbis have ruled that in light of the obligation to save life as established in Jewish law, priority in medical treatment should be given to the one who is in a life-threatening situation (Maimonides 1954, “Laws of the Murderer,” 1:14; Auerbach 1999: vol. 2 (B-C), Ch. 86). That is, it is preferable to treat the one likely to obtain the maximum benefit from the treatment. Some of the rabbis who adopted this rule relied on two opinions: that of Rabbi Avraham Yishiyahu Karelitz (Eastern Europe and Israel, 1878–1953), a leading twentieth-century halachic arbiter and one of the architects of the ultra-Orthodox way of life in Israel, and that of the Chief Rabbi of Israel from 1964 to 1972, Rabbi Isser Yehuda Unterman (1886–1976). Both concluded that if there is a resource that does not belong to either person who needs it, it should be given to the one who will derive the greatest benefit (Karelitz 1996; Hazon Ish, sec. 20). According to Rabbi Karelitz, one should give preference to one person, rather than extend the lives of both for a short time.

Rabbi Unterman also ruled the same way in the case of a doctor who was needed by many patients and could not get to all of them. To meet “the obligation … to give the patients enough care to heal them,” he should heal them for a long and normal life and not split himself among all of them such “that each receives only temporary relief” (Unterman 1991, 316). As, in that case, the medicine was owned by a private person, Rabbi Unterman added: “Since he is not ill now, one cannot say ‘your life takes precedence,’ since it is not a question of saving life.”

Rabbi Eliezer Waldenberg (1915–2006) was a major twentieth-century halachic arbiter, a member of the Rabbinical High Court, and the rabbi of the Shaarei Tzedek Hospital in Jerusalem and was known primarily for his Responsa in connection with medicine and halacha. In his book of Responsa *Tzitz Eliezer* (Waldenberg 1984, part 9, sec. 28), he described a case where there were two patients requiring the same medicine and the hospital did not have enough for both of them. Rabbi Waldenberg ruled that in this case it was not possible to follow the guidance of Rabbi Akiva that “your life takes precedence” since neither person was the owner of the medicine.

What should the ruling be in a case of many patients and a single dose of medicine such as once occurred in Hadassah Hospital, Jerusalem? The hospital asked for advice from the sitting Chief Rabbi of Israel (1936–1959) Rabbi Yitzhak Isaac Herzog (1888–1959). A description of the case was presented by one of the doctors who was present during a discussion at a meeting of one of Israel’s parliament’s committees:

Many years ago, at the end of the Second World War, there occurred at Hadassah Hospital a most interesting case: the illness “bacterial meningitis” had spread, with a mortality rate one of 100% until penicillin was discovered. When the drug arrived, which was only enough to treat one patient, there were 450 patients in the hospital. The dilemma arose as to who should be treated. The youngest of the child patients or an older person? Someone who held a senior position or someone unemployed? We contacted the then Chief Rabbi, Rabbi Herzog of blessed memory, to help us to make a decision. In the end it was decided that the doctor would treat the first patient he came upon in the department. How can we decide whose life is worth more? (Knesset’s Labour and Welfare Committee 1987, 5)

That is to say, as there was only enough of the drug for one patient, the entire supply would be used for the patient that the doctor saw first, as it was impossible to decide who had priority over whom. Rabbi Herzog’s approach was that everyone is equal and that no one has the authority to decide that the life of one individual is more important than the life of another.

In any case, each of the rabbis did not deal with the identity of the patient or his/her criminal past but examined it only against the background of his/her medical condition.

## Biblical Injunctions Regarding the Concern for the Medical Well-Being of One’s Fellow Man as a Duty to G-d

Jewish tradition sees the obligation to deal with a pandemic from two perspectives: the religious aspect, which calls for self-correction, fasting, and prayer, and the medical and civil challenges (Rashi 2020). Maimonides (Moses ben Maimon [1138–1204]), one of the most important legal arbiters in Jewish history, noted:

These are the troubles of the community about which we fast and sound [trumpets] … about a plague … About a plague—how is this? What is a plague? A city that has 500 footmen and three of them died in three days—one after [the other]—this is surely a plague. If they were eliminated in one day or in four days, it is not a plague. If there were a thousand and six of them were eliminated in three days—one after [the other—this is] a plague. If they were eliminated in one day or in four days, it is not a plague. And so forth, according to this calculation. And women, minors, and old men that have stopped from working are not in the count of the people of the province regarding this matter. [If] there was a plague in the Land of Israel, the rest of the exiles of Israel should fast about them. If there was a plague in a province and there are caravans coming from it to another province—both of them should fast, even though they are far from one [another]. (Maimonides 1954, “Laws of Fasts”, 2: 1, 5–6)

The demand for prayer and fasting in light of a plague that has harmed one’s own community or people in other places reflects the understanding that there is a mutual responsibility to care for others wherever they are.

However, epidemics are not subject solely to theological considerations but involve a series of medical and social imperatives that are strengthened by religious norms. Maimonides, who was also a philosopher and physician, did not stop with just the religious commandment to pray and fast during a plague. He also insisted that there is a halachic obligation not only to treat disease but also to prevent it from spreading and that doing so requires medical and social concerns:

Seeing that the maintenance of the body in a healthy and sound condition is a G-d-chosen way, for, lo, it is impossible that one should understand or know aught of the divine knowledge concerning the Creator when he is sick, it is necessary for man to distance himself from things which destroy the body and accustom himself in things which are healthful and life-imparting. (Maimonides 1954, “Hilchot De’ot”, 4:1)

That is to say, every individual must look after his/her own health so as not to become ill. Thus, as a doctor, he noted that people should not eat unhealthy foods and should protect themselves from catching a cold and from all other things that can harm the body.

Maimonides basically argued that one has to avoid getting sick because an unhealthy person cannot worship G-d in a suitable fashion, nor can such a person study properly. As both a physician and a religious leader, he argued that one has a duty to protect oneself from activities that damage one’s health both because health is a good in and of itself and it is difficult for an unhealthy person to fulfil religious duties. He found his proof text for these arguments within the following biblical injunctions: “Only be careful, and watch yourselves closely” (Deuteronomy 4:9) and “Therefore watch yourselves very carefully” (Deuteronomy 4:15). Further, in the Babylonian Talmud (1990–2012) (Ta’anit 22B, based on Genesis 2:7), we read, “the man became a living being”—the soul I gave you—keep it alive. Maimonides also noted, “If any obstacle involves a danger to life, it is a positive command to remove it and to be aware of it, and to be particularly careful, as it says: ‘“Only be careful, and watch yourselves closely’” (Maimonides 1954, “Hilchot Murderer,” 11–12). Clearly, the import of these words is that the duty of maintaining health is a religious obligation of a man toward his G-d.

## The Duty to Heal as Part of One’s Responsibility to Others

Maimonides also expanded the prohibition of the duty to heal into a general guideline: “The sages have prohibited many things because they are dangerous to life. If anyone disregards them and says: ‘What claim have others on me if I risk my own life?’ or ‘I do not mind this,’ he should be lashed for disobedience” (Maimonides 1954, “Hilchot Murderer,” 11:5). That is to say, according to Maimonides, every instruction issued concerning taking care to protect the well-being of the body and its health is an indication that it is forbidden for an individual to put himself and others in danger or to treat the instructions he has been given with contempt. If a person ignores those instructions, he should be punished by flogging at the discretion of the rabbinical court because obeying them is part of one’s religious duty toward others.

Maimonides’ injunction draws on biblical verses that declare that a person must not limit himself to worrying about his own health but should also be concerned about the health of his fellow. For example, as in Leviticus 19:16, “Do not do anything that endangers your neighbour’s life,” and Leviticus 19:18, “Love your neighbour as yourself.” These biblical injunctions are rules and specific instructions as to how to act in particular situations. Thus, in Maimonides (1954, “Hilchot Mourning,” 14:1) he wrote: “You shall love your neighbour as yourself” (Leviticus 19:18); that is, whatever you would have others do for you, do for your brothers. In other words, love for another is expressed by a series of deeds that in fact every person would wish would be done for him, and he is required to do them himself.

Some halachic arbiters understood that the biblical prohibitions also had medical and social significance. For example, the ruling of Rabbi Judah ben Samuel of Regensburg (1150–1217), also known as Rabbi Judah HaHasid, or Judah the Pious, who was a leader of the Hasidei Ashkenaz, a movement of Jewish mysticism in Germany, is especially relevant. Among other adjudicators, Rabbi Judah HaHasid wrote that the biblical commands to love one’s neighbour and not to place stumbling blocks before him are applicable when a person is sick and might infect others:

“Do not ... put a stumbling block in front of the blind” (Leviticus 19:14), that a person with a contagious skin disease should not bathe with another Jew unless he informed him first, as it is written (op. cit. 18) “Love your neighbour as yourself” (op. cit. 19) and “Do not do anything that endangers your neighbour’s life” (op. cit. 16). (Judah ben Samuel 2002, sec. 773)

That is to say, a sick person is obliged to warn those around him against the possibility that he might transmit the infection, an obligation that derives directly from biblical commandments. Moreover, Rabbi Judah HaHasid’s words are completely in line with the halachic rulings of the Baalei Tosafot, French and German rabbis from the twelfth century to the fifteenth-century, who wrote critical and explanatory glosses (questions, notes, interpretations, rulings, and sources) on the Talmud, collectively called Tosafot (additions). The Tosafot are important for the practical application of Jewish law because that law depends on how the Talmud is understood and interpreted.

In one of their commentaries on the Babylonian Talmud, the Tosafot wrote: “A person must make greater effort not to harm someone else than not to be harmed himself” (Babylonian Talmud, Baba Kama 23A). It is important to understand that this interpretation from medieval times is based on an understanding formulated over the years among halachic arbiters about the obligation to preserve health in general, to guard personally against illness, and to be extremely careful during an epidemic.

Some will say that the obligation to take care of the other’s health is relevant only for those who do not break the law and that felons are not entitled to the concern and care that should characterize a decent community. Jewish ethics holds a different view, which I discuss in the following pages.

## Attitude Toward the Criminal and the Prisoner in Jewish Ethics

The approach of Jewish ethics to human rights derives first and foremost from the principle that appears at the start of the Book of Genesis in its description of the beginnings of human history and the creation of man:

Then G-d said, “Let us make mankind in our image, in our likeness, so that they may rule over the fish in the sea and the birds in the sky, over the livestock and all the wild animals, and over all the creatures that move along the ground.” So G-d created mankind in his own image, in the image of G-d he created them; male and female he created them. (Genesis 1:26–27)

The revered first-century Jewish Sage Rabbi Akiva explained this: “Man is beloved because he was created in the Image. An extra fondness was granted him because he was created in the Image, as it says: ‘Because in G-d’s Image man was made’” (Neusner 1991, Ethics of the Fathers, 3:14).

According to the principles of Jewish ethics, the concept of the creation of man in G-d’s image mandates honourable behaviour even toward prison inmates. Therefore, individuals incarcerated on account of their crimes, although they might arouse rejection and disgust, are still people and are entitled to be treated humanely, according to the norms of an advanced society, as a civilized society would wish to be.

Jewish ethics starts with the assumptions that one must respect the rights of a prisoner and that those rights should only be limited to the degree required by the imprisonment itself. Similar to all “human rights,” the starting point of Jewish ethics is that the preservation of a prisoner’s dignity and well-being is not a “right” but a societal obligation. Jewish ethics is strict about a prisoner’s honour not because it is lenient about a punishing a criminal for his crime or unconcerned about the need to protect society from harm at his hand or to deter other potential wrongdoers, but because the issue is one of respect because mankind was created in “the image of G-d.”

In Deuteronomy 25: 1–3, we read:

If there be a controversy between men, and they come unto judgment, and the judges judge them, by justifying the righteous, and condemning the wicked, then it shall be, if the wicked man deserves to be beaten, that the judge shall cause him to lie down, and to be beaten before his face, according to the measure of his wickedness, by number. Forty stripes he may give him, he shall not exceed; lest, if he should exceed, and beat him above these with many stripes, then thy brother should be dishonored before thine eyes.

The Midrash halacha was the ancient Judaic rabbinic method of Torah study that expounded upon the traditionally received 613 commandments by identifying their sources in the Hebrew Bible 2016 and interpreting these passages as proofs of the laws’ authenticity.^4^ The Midrash *Sifre* 2014, classical Jewish legal biblical exegesis, based on the biblical books of Numbers and Deuteronomy notes: Rabbi Chanania ben Gamliel said that the Bible calls the defendant who harms his friend “evil,” but as soon as the defendant is punished—the Bible calls him “your brother” (Sifre, 286: 304).

That is to say, as long as a person is accused of a certain crime, the Bible deems him wicked, and the attitude toward him must be seriously critical, but after he has been punished he has to be considered as a brother. Brotherhood in regard to a prisoner demands that he not be harmed beyond the punishment meted out for his crime. In this connection, two fundamental principles of the laws of punishment in Jewish jurisprudence can be noted. According to Maimonides:

All these things [methods of punishment] as they are deemed applicable by the Jewish judge and as the circumstances require; and everything he does should be in Heaven’s name and he should not disdain respect for people .... He should be careful not to destroy their self-respect, but should only enhance respect for G-d. (Maimonides, “Laws of the Sanhedrin,” 24:10)

The second principle comes from a Talmudic story based on Psalm 104:35— “But may sins vanish from the earth and the wicked be no more”—recounted about Rabbi Meir and his wife, Bruria, in the second century:

Hoodlums in Rabbi Meir’s neighbourhood were giving him a great deal of trouble and Rabbi Meir wanted them to die. Bruria his wife said to him: “What is your reason (that you prayed that they should die)?” [He answered:] “Because of the verse ‘May sins vanish.’ [She asked:] “Does it say sinners? No, it says sins! And more: You should read the end of the verse: ‘and the wicked be no more,’ as it says, ‘May sins vanish and the wicked be no more’! Therefore, you should ask mercy for them and pray that they will repent ‘and that the wickedness will be no more.’” He prayed for them and they repented. (Babylonian Talmud, Brachot 10A).

That is, a good person does not pray for the deaths of criminals but rather that they will correct their ways. Thus, punishment is not meant to focus on executing criminals but rather on reforming them.

There are sometimes crimes for which society must impose the death penalty. Even so the approach toward such felons in Jewish ethics is that the prisoner is nonetheless a member of society, and the words “And you shall love your neighbour as yourself” (Leviticus 19:18) apply to him as well. This verse is a well-known guiding principle and is generally considered a directive for interaction between people regardless of who they may be. The Sages interpreted that to mean that one must act respectfully even toward a sinner condemned to death: “Choose him a good death” (Babylonian Talmud, Sanhedrin 45A). That is to say, even if someone carried out so serious an act as to be sentenced to death, one should ensure as easy a death as possible, free of excess humiliation and pain. The Talmud there also adds: “Somebody about to be executed should be given frankincense to drink^5^ in a glass of wine so that he will become senseless, as it says, ‘Let beer be for those who are perishing, wine for those who are in anguish’” (Proverbs 31:6); further the Talmud notes: “Worthy women of Jerusalem volunteered to bring them [the wine]” (Babylonian Talmud, Sanhedrin, 43A).

In other words, the obligation to be concerned about one’s fellow does not differentiate between the righteous and the evil, and the Talmud praises the women who volunteered to carry out a sensitive and humane act of kindness toward criminals condemned to death and calls them, “worthy women of Jerusalem.” (Rashi and McCombs, 2017).

## Conclusion

The weakness of prisoners’ struggle for their rights derives from two causes: first, because their freedom, the greatest of man’s rights alongside the right to life itself, has been taken from them and they are in the custody of the state; second, because they generally lack a “lobby” in the political and social sense, and there is no one to speak on their behalf to represent their interests.

According to Jewish ethics, withholding a vaccination is not part of the designated punishment, so one should not differentiate between incarcerated individuals and men and women outside the prison walls. There is an obligation to all of them to guard their health. Accordingly, all are equally entitled to the vaccination against COVID-19 and there is a moral and religious obligation to care for them equally (in accord with professional priorities established by the Health Ministry of each country).

In the Talmud, we read “A prisoner cannot release himself from prison” (Babylonian Talmud, Berachot 5B); that is, someone who has become used to the hard life of imprisonment is incapable of freeing himself from incarceration and its effects and needs assistance to do so. The understanding that the provision of vaccinations is not a prisoner’s “right” but has to be defined as a moral obligation (alongside medical necessity and the public interest) is not merely a question of semantics but should also inform its early and equitable implementation.

## Footnotes

1 For tracking of how many people were sickened and killed by COVID-19 in prisons and how widely it spread across the country and within each state in the United States, see: https://www.themarshallproject.org/2020/05/01/a-state-by-state-look-at-coronavirus-in-prisons.

2 The duty to rescue is, of course, much more complicated: In Israel, there is a law that reads “[one] will not stand on the blood of another” (1998). In contrast to the good Samaritan law that deals with protection against civil lawsuits, Israeli law mandates active assistance at need. In Israel, the 1998 law “You will not stand on the blood of a neighbour” (based on Leviticus 19:16 as interpreted in the Babylonian Talmud, Sanhedrin 73A), was enacted following a private member’s bill by MK Hanan Porat. When it was brought to a final vote, MK Porat justified his initiative: We would not need this law if we really were in a situation where the natural morality, according to which a person who sees another person in distress before his eyes would not hesitate, would take and interest, and assist, even if it cost him a lot of money and if it puts him at risk. We thought, however, because of very unfortunate cases in recent years and in light of the desire to establish a norm that anchored in Jewish morality, based on “you will not stand on the blood of a neighbour,” that one cannot stand aside when one sees a person in danger: A Jew or Gentile, an adult or a minor, a man or a woman, one must reach out to him and save him (Knesset Remarks 1998, 111). Owing to its uniqueness—the imposition of a duty to do, which is fundamentally a moral stance—the law was enacted separately from the Penal Code: The law is not only saying what is forbidden but is also a statement regarding the right thing to do. It is common to see the scope of the duty it imposes as minimal: a mandate to help one another and a notification to the authorities to assist wherever and whenever they are asked (Rashi 2021). Such laws are common in Europe but are rarely found in the Anglo-American legal tradition. In the European tradition they are attributed to Judeo-Christian ethics and are known as “Good Samaritan Laws,” after a parable in the Gospel according to Luke: two men ignored another man who had fallen victim to theft and violence, but the Good Samaritan took care of him. The obligation to rescue is not found in English law. In the United States, two trends can be identified. In most states, the obligation does not exist, although many laws encourage rescue and provide immunity, relief, and compensation to the rescuer. In Vermont, Minnesota, Rhode Island, and Wisconsin, the rescue obligation has been established owing to public pressure following rape cases in which witnesses did not intercede. For more on the importance of the individual’s life and the balances required in regard to saving the individual and the many from an ethical and Jewish point of view, see Rashi 2018.

3 The text is in the Kaufman Manuscript and in the Parma Manuscript of the Mishnah, as well as in printed versions of the Jerusalem Talmud, without the words “from Israel.” But in various manuscripts and printed versions of the Babylonian Talmud and in the printed versions of the Mishnah, the words “from Israel” appear, and the sentence reads: “he who saves one life from Israel saves a whole world.” In his *Mishneh Torah*, Maimonides quotes a version of the Mishnah in which the word “Israel” is replaced by “in the world,” for example, in the manuscript of Yah. Ms. Heb. 2. In most versions, the word “from Israel” does not appear, but there is one that reads “he who saves one life from Israel saves a whole world.” The absence or presence of the words “from Israel” has provoked lengthy discussions: is it a humanistic statement, which attaches importance to the life of each and every person created in the image of G-d or is it a national statement, which attaches importance only to the life of a person from Israel (i.e., a Jew). This discussion is always preceded by the question about the wording of the original version, and whether it is possible that the variations are distortions created over the years. The late Ephraim Elimelech Urbach, a professor of Talmud at the Hebrew University of Jerusalem and the president of the Israel Academy of Sciences and Humanities. presented a clear position on this issue: Reading the article in the Mishnah in its entirety ... points in the direction of the wording that does not include “from Israel,” since it is a conclusion about the importance of human life from the fact that the first man was created alone. ... However, the Mishnah answers the question “how do you interrogate witnesses.” These witnesses were from Israel and they came to testify about one Jew who murdered another Jew. A distinction must therefore be made between utilizing the text to teach morality and its use in interrogating witnesses (Urbach 1971: 269).

4 According to Jewish tradition, the Ten Commandments were given to Moses at Mount Sinai over 3000 years ago in the presence of the entire Israelite nation (600,000 adult males). The full Torah, written by Moses toward the end of his life, contains 613 commandments. Around these commandments and accompanying elaborations and clarifications (Written Law) there evolved an Oral Law comprising rabbinic discussions and arguments over the ensuing centuries that ultimately coalesced into the Halakhah. In the second century of the Common Era, the period in which the first major codification of Jewish Law, the Mishnah, was written, rabbis set up a major centre of scholarly religious learning to facilitate continuation of the halakhic tradition. In the ensuing eighteen centuries, generations of religious leaders living in many countries around the world under the influence of various religiously oriented civilizations (Catholic, Eastern Orthodox, Moslem, Protestant, etc.) continued to broaden and further clarify and codify the Halakhah, which is a praxis-based code of law (i.e., legal principles are derived from specific problems and issues that arise during daily life, much like English Common Law). Thus, during the past 1500 years tens of thousands of common-man “questions” and local rabbinical “answers” (in Hebrew: “Shut”) have “clarified” the Halakhah, thereby naturally developing what has come to be called “Responsa literature.” Every so often, owing to the unwieldiness of such a huge corpus, major rabbinical commentators have taken it upon themselves to “codify” the law in some systematic and quasi-authoritative fashion (Rashi 2012, 97).

5 The use of frankincense as an anti-anxiety agent was known in many ancient societies. The long history of its use has led researchers to seek to identify its active ingredients and the way it works. For further information about the psychoactive effect of frankincense, see Moussaieff, et al. 2008.
